# Foot kinematics in patients with two patterns of pathological plantar hyperkeratosis

**DOI:** 10.1186/1757-1146-4-7

**Published:** 2011-02-09

**Authors:** Andrew H Findlow, Christopher J Nester, Peter Bowker

**Affiliations:** 1 1Centre for Health, Sport and Rehabilitation Sciences Research, School of Health, Sport and Rehabilitation Sciences, University of Salford, Salford M6 6PU, England, UK

## Abstract

**Background:**

The Root paradigm of foot function continues to underpin the majority of clinical foot biomechanics practice and foot orthotic therapy. There are great number of assumptions in this popular paradigm, most of which have not been thoroughly tested. One component supposes that patterns of plantar pressure and associated hyperkeratosis lesions should be associated with distinct rearfoot, mid foot, first metatarsal and hallux kinematic patterns. Our aim was to investigate the extent to which this was true.

**Methods:**

Twenty-seven subjects with planter pathological hyperkeratosis were recruited into one of two groups. Group 1 displayed pathological plantar hyperkeratosis only under metatarsal heads 2, 3 and 4 (n = 14). Group 2 displayed pathological plantar hyperkeratosis only under the 1^st ^and 5^th ^metatarsal heads (n = 13). Foot kinematics were measured using reflective markers on the leg, heel, midfoot, first metatarsal and hallux.

**Results:**

The kinematic data failed to identify distinct differences between these two groups of subjects, however there were several subtle (generally <3°) differences in kinematic data between these groups. Group 1 displayed a less everted heel, a less abducted heel and a more plantarflexed heel compared to group 2, which is contrary to the Root paradigm.

**Conclusions:**

There was some evidence of small differences between planter pathological hyperkeratosis groups. Nevertheless, there was too much similarity between the kinematic data displayed in each group to classify them as distinct foot types as the current clinical paradigm proposes.

## Background

Clinical diagnosis and orthotic management of mechanically related foot disorders is founded on a the generally accepted Root *et al *[1,2] paradigm of foot function. This paradigm was developed in response to a clinical need for a conceptual framework to classify and explain foot pathologies. Despite a lack of kinematic data supporting such concepts, 'mobile' and 'rigid' foot types are central to the paradigm. The belief is that the mobile foot type is characterised by a more everted heel and a lower medial arch profile compared to the rigid foot type. The assumed differences in foot kinematics between the mobile and rigid foot types are associated with similarly distinct patterns of load distribution under the forefoot. For the mobile foot type pressure is primarily located under the second and third metatarsal heads. This is said to be a consequence of medial distribution of load under the forefoot due to rearfoot eversion and dorsiflexion of the first metatarsal head relative to the second. This leaves the second metatarsal head relatively "exposed" and bearing substantial load, with progressively less load on the third, fourth and fifth metatarsals. The dorsiflexion of the first but not the second metatarsal is said to be due to its greater mobility and recent data lends some credibility to this [3,4]. Thus, the mobile foot is thought to be associated with greatest load on metatarsal head two with progressively less on three and four.

In contrast, in the rigid foot type the relatively less pronated, or supinated rearfoot position, leads to more load under the lateral rather than medial forefoot. In further contrast to the mobile foot type, the mobility of the lateral forefoot in the rigid foot type is reduced (because the foot is more 'rigid') and the fifth metatarsal does not dorsiflex under the increased lateral loading. It thus bears substantial loads. The relatively reduced load under the medial forefoot is thought to provide less resistance to the windlass mechanism, that plantarflexes the first metatarsal as the hallux dorsiflexes during terminal stance. The subsequent greater first metatarsal plantarflexion compared to the mobile foot type increases the height of the medial arch. The relatively plantarflexed position of the first metatarsal is believed to result in relative unloading of the second and third metatarsals and leave the first metatarsal bearing substantial loads. Thus, the rigid foot type is associated with greatest load under the metatarsal heads one and five.

One proposed clinical manifestation of the hypothetical differences in foot kinematics and load distribution under the forefoot between mobile and rigid foot types, is the development of distinct patterns of pathological plantar hyperkeratosis (PPH). The thickening of the stratum corneum in response to repeated high levels of load is generally acknowledged as associated with an increased plantar pressure [5-8]. Thus, it is supposed that the pattern of PPH distribution under the metatarsal heads will reflect the pattern of load distribution under the forefoot, which according to the clinical paradigm, is associated with distinct patterns of foot kinematics and the 'mobile' and 'rigid' foot types. An important consequence of the formation of PPH is that it is acknowledged clinically to be a precursor to plantar foot pathologies in high-risk category patients, for example neuropathic plantar foot ulceration in people with diabetes.

There are clearly a great number of assumptions in this popular clinical paradigm of foot function. Rather than break the paradigm down into its constituent assumptions and evaluate each in isolation, in this study we chose the take a pragmatic approach to evaluating the foot type concepts within the paradigm. According to the paradigm, patterns of foot pressure and PPH lesions should be associated with distinct rearfoot, mid foot, first metatarsal and hallux kinematic patterns. Our aim was to investigate the extent to which this was true.

## Methods

**Figure 1:**
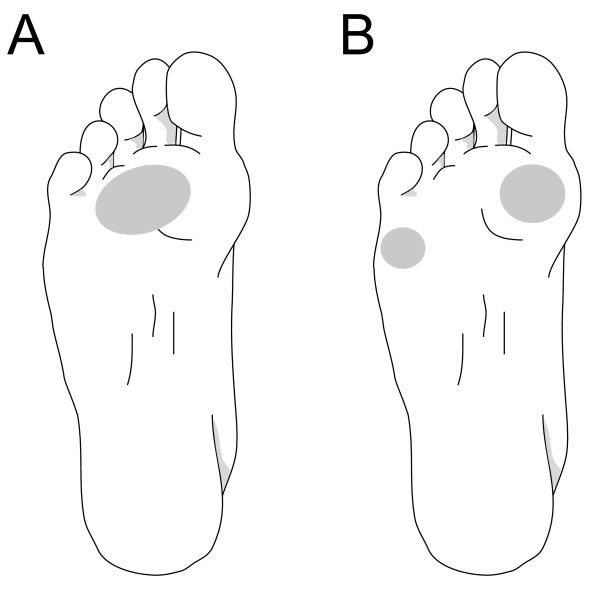
**Example of callus patterns**. A - Example of callus pattern for group 1 - under metatarsal heads 2, 3 and 4; B - Example of the callus pattern for group 2 - under metatarsal heads 1 and 5.

Following ethical approval (University of Salford Ethics committee) 27 subjects (table 1) who attended the University Podiatry clinic every 4-8 weeks for debridement of plantar callus were recruited and gave informed consent. The inclusion criterion was one of two types of forefoot PPH pattern. Group 1 displayed PPH only under metatarsal heads 2, 3 and 4. Group 2 displayed PPH only under the 1^st ^and 5^th ^metatarsal heads (n = 13). PPH (callus) was a distinct area of thickened and hardened upper layer of the skin having distinct boundaries with normal skin, and a regular oval outline (Figure 1). Whilst no measure of foot posture or type was used, anecdotally, subjects in Group 1 had a physical appearance of *pes planus *(low medial arch profile) and those in Group 2 displayed *pes cavus *(high medial arch profile). These were consistent with the Root paradigm. None of the subjects displayed heloma durum. All subjects showed the same PPH pattern on both feet, except for three subjects who displayed the pattern under the left forefoot only. Thus, total sample was 24 limbs from group 1 (11 right, 13 left), 27 limbs from group 2 (13 right, 14 left). All subjects had negative history of lower limb injury or systemic disease (e.g. diabetes, rheumatoid arthritis).

**Table 1:** subject descriptive statistics

		n	Mean	Std. Dev	Std. Error	95% Confidence Interval for Mean		Min	Max	Significant difference
									
						Lower Bound	Upper Bound			
AGE	PPH 234	14	46.48	15.92	4.25	37.29	55.67	22.73	76.97	0.352
	
	PPH 1 and 5	13	52.44	16.69	4.63	42.35	62.52	27.18	75.21	
	
	Total	27	49.35	16.29	3.13	42.92	55.78	22.73	76.97	

WEIGHT	PPH 234	14	82.86	13.63	3.64	74.99	90.73	52.40	109.8	0.120
	
	PPH 1 and 5	13	74.31	13.92	3.86	65.90	82.72	46.00	96.40	
	
	Total	27	78.74	14.19	2.73	73.13	84.35	46.00	109.8	

HEIGHT	PPH 234	14	1.70	0.10	0.03	1.64	1.75	1.58	1.87	0.034
	
	PPH 1 and 5	13	1.62	0.07	0.02	1.58	1.66	1.52	1.71	
	
	Total	27	1.66	0.09	0.02	1.63	1.70	1.52	1.87	

**Figure 2:**
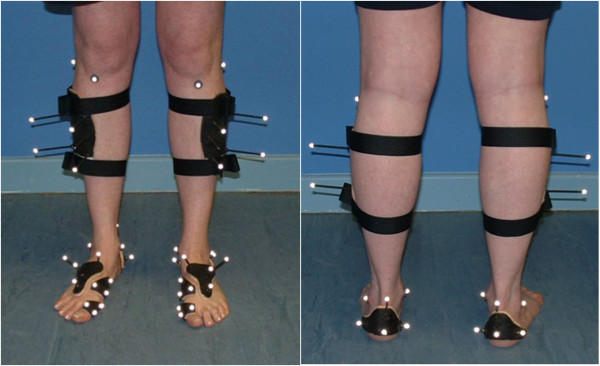
**Markers located on 5 plates**. To define co-ordinate frames for the leg, heel, mid foot, first metatarsal and hallux. Markers on the skin of the shank were used to align the tibial LCF to the shank anatomy.

Foot kinematics were measured using reflective markers on the leg, heel, midfoot, first metatarsal and hallux [9-13] (figure 2) and 100 Hz infrared cameras [14]. The performance of the six-camera Qualisys ProReflex system was tested prior subject data collection to optimise the position of the cameras for the 6 mm markers used in the study. The accuracy and precision (RMS error of 0.33 mm, SD 0.31 mm) of the Qualisys ProReflex system using this configuration are better than some previous results (e.g. Ehara *et al *[15] RMS between 0.9 mm and 6.3 mm, SD 0.8 mm to 6.0 mm). Each subject was allowed a reasonable period of time to become familiar to the gait lab environment and the marker clusters before ten gait trials at a self-selected pace were recorded (walking speed was not measured). Local co-ordinate frames (LCF) were defined for each segment. For the tibia anatomical markers on both malleoli, fibula head and tibial tuberosity were used to align the LCF relative to the technical markers on the mid shin [9-11]. For the heel and midfoot the LCF was set parallel to the global system when in relaxed standing. For the first metatarsal and hallux, reflective markers were positioned on the plates to enable the anterior/posterior (x) axis to follow the approximate long axis of the metatarsal and hallux respectively. The medial/lateral axes were 90° to the x-axis and parallel to the supporting surface. Rotations between distal and proximal adjacent segments were calculated using Euler rotation sequence z x y. Data were normalised to 0-100% of stance and averaged across ten trials. The reference position (0 degrees) was the foot position when the subject stood upright (figure 2). Other studies have used a subtalar "neutral" position [16-18], which lacks validity (has no proven functional meaning) and has been shown to be more subjective [19-23].

The parameters used to characterise foot kinematics in the two groups were directly related to the clinical paradigm and enabled a comprehensive exploration of foot kinematics. These were: the *angular position *of each joint in each plane at each of 7 gait events: Heel Contact (HC), Foot Flat (FF), Ankle Neutral (AN), Heel Off (HO), Maximum Ankle Dorsiflexion (MAD), Maximum Toe Dorsiflexion (MTD), and Toe Off (TO). In addition, the *range of motion *(ROM) at each joint and in each plane of motion was derived during HC to FF, FF to AN, AN to HO, HO to MAD and HO to TO. Finally, the timing of FF, AN, HO, MAD and MTD were derived (% of stance).

Ankle neutral was defined as the time at which the sagittal plane leg/heel data was 0°. Foot marker velocity and displacement data were used to detect HC, FF, HO and TO [24-27]. The vertical velocity of the origin and x and y-displacement of the heel LCF was used to detect HC and HO respectively. Y-displacement of the origin of the forefoot LCF was used to detect FF. x-axis displacement of the origin of the hallux LCF was used to detect TO. Differences (error in seconds) between force plate and foot kinematic data definitions of these events were tested in a pilot study on 11 subjects and are detailed in table 2. The mean errors are no greater than 0.024 seconds, or <3% of stance.

**Table 2:** Mean (SD) error in detection of foot contact events (seconds)

	Contact event			
	***Heel contact***	***Foot flat***	***Heel off***	***Toe off***

Mean error (seconds)	0.007 (0.005)	0.021 (0.020)	0.024 (0.022)	0.016 (0.015)

Differences between groups were tested using ANOVA. However, the data could be additionally classified using side (i.e. differences between left and right limb), to determine if any variances in these data were due to interaction or covariance of these factors; ANOVA was computed with 'Two-Factor Interactions' i.e. 'PPH group' and 'side'.

## Results

**Figure 3:**
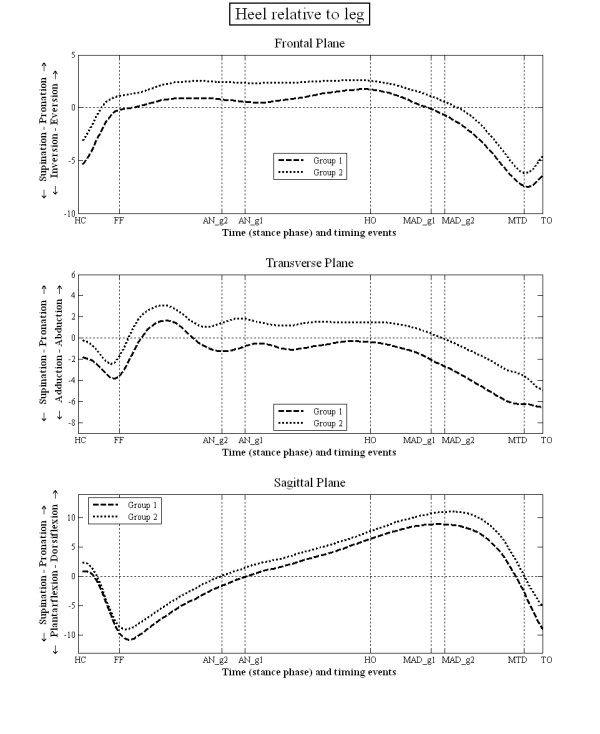
**Motion of heel LCF relative to leg LCF during stance phase of gait**.

**Figure 4:**
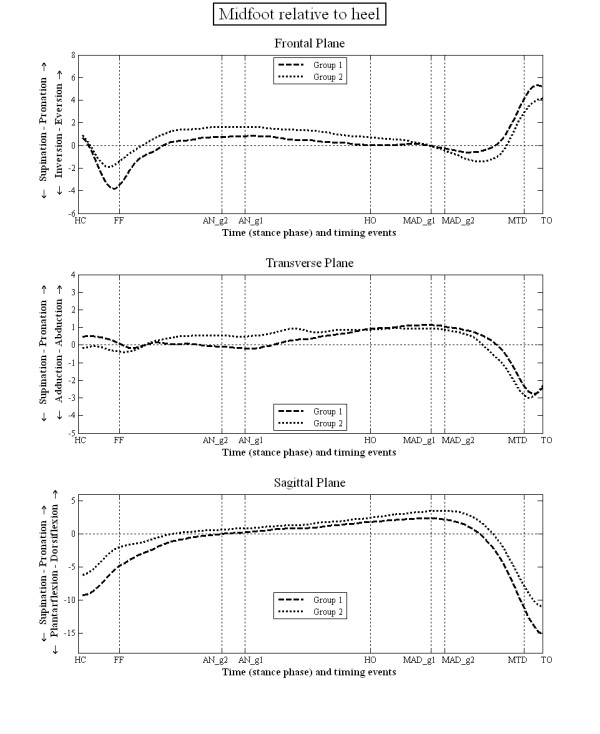
**Motion of midfoot LCF relative to heel LCF during stance phase of gait**.

**Figure 5:**
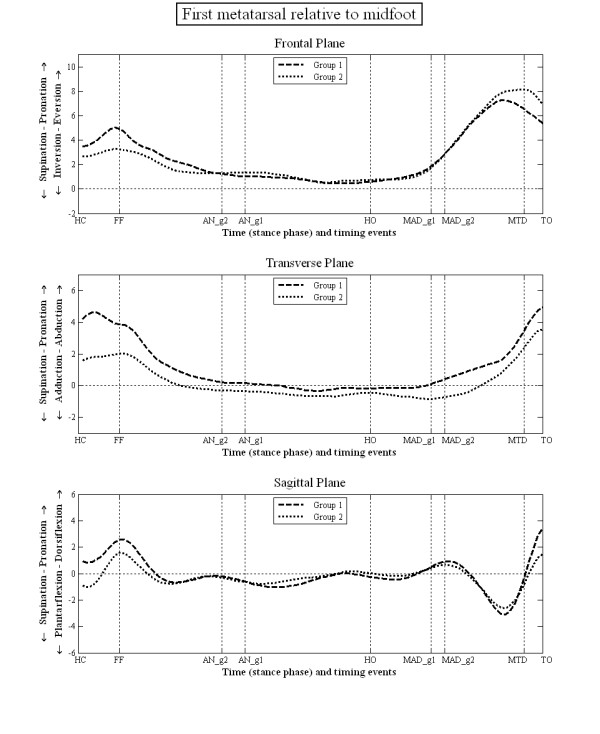
**Motion of 1st metatarsal LCF relative to midfoot LCF during stance phase of gait**.

**Figure 6:**
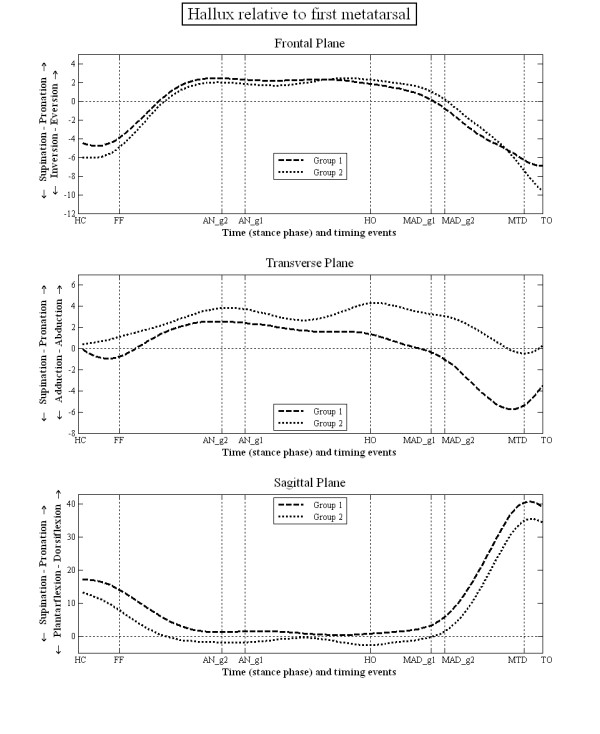
**Motion of hallux LCF relative to 1st metatarsal LCF during stance phase of gait**.

The mean kinematic data during stance for each group are illustrated in figures 3, 4, 5 and 6. There were no statistically significant differences in the PPH groups based on the side i.e. between left and right limbs. However, there were statistically significant differences between group 1 and 2 in terms of the relative position and ROM at the joint studied (tables 3 and 4). Group 1 displayed greater heel inversion at heel contact (-5.4° compared to -3.1°), greater heel plantarflexion at foot flat (-9.2° compared to 3.3), but less heel dorsiflexion at the time of heel off and time of maximum ankle dorsiflexion (6.7° compared to 8.9°). Group 1 displayed greater heel plantarflexion at toe off (-9.0° compared to -5.1°). In the transverse plane, the heel in the feet of group 1 was less abducted at the time of ankle neutral (-1.1° compared to 1.5°), heel off (-0.4° compared to 1.5°) and the time of maximum ankle dorsiflexion (-2.1° compared to -0.1°). The heel was also more adducted at the time of maximum hallux dorsiflexion (-6.1° compared to -3.8°).

**Table 3:** Significant differences between the PPH groups in angular displacement for the ankle/subtalar joint complex and midtarsal joint p ≥ 0.05.

Joint/Complex	Gait event	Cardinal Body Plane	Group 1 (PPH 2, 3 and 4)			Group 2 (PPH 1 and 5)		
			
			mean	St. Dev	95% CI (upper/lower)	mean	St. Dev	95% CI (upper/lower)
Leg/Heel	HC	frontal	-5.4°	2.7°	-10.7°/-0.17°	-3.1°	2.9°	-8.8°/2.6°
	
	FF	sagittal	-9.2°	2.4°	-13.9°/-4.5°	3.3°	3.3°	-3.2°/9.8°
	
	AN	frontal	0.9°	1.7°	-2.4°/4.2°	2.4°	2.3°	-2.1°/6.9°
		
		transverse	-1.1°	2.6°	-6.2°/4.0°	1.5°	3.2°	-4.8°/7.8°
	
	HO	transverse	-0.4°	2.3°	-4.9°/4.1°	1.5°	3.5°	-5.4°/8.4°
		
		sagittal	6.7°	2.5°	1.8°/11.6°	8.9°	3.3°	2.4°/15.4°
	
	MAD	transverse	-2.1°	3.1°	-8.2°/4.0°	0.1°	3.7°	-7.2°/7.4°
		
		sagittal	9.3°	2.9°	3.6°/15.0°	11.5°	3.3°	5.0°/18.0°
	
	MTD	transverse	-6.1°	3.7°	-13.4°/1.2°	-3.8°	3.9°	-11.4°/3.8°
	
	TO	sagittal	-9.0°	4.4°	-17.6°/-0.4°	-5.1°	5.2°	-15.3°/5.1°

Mid foot/Heel	HC	sagittal	-9.3°	3.1°	-15.4°/-3.2°	-6.2°	2.4°	-10.9°/-1.5°
	
	FF	frontal	-3.4°	2.5°	-9.0°/0.8°	-1.6°	3.0°	-8.3°/3.5°
		
		sagittal	-5.7°	2.4°	-10.4°/-1.0°	-2.9°	3.2°	-9.2°/3.4°
	
	MTD	sagittal	-10.8°	5.0°	-20.6°/-1.0°	2.9°	2.9°	-2.8°/8.6°
	
	TO	sagittal	-15.3°	4.9°	-24.9°/-5.7°	-11.1	3.2°	-3.1°/9.5°

First metatarsal/Mid foot	HC	transverse	4.2°	4.2°	-4.0°/12.4°	1.6°	3.0°	-4.3°/7.5°
		
		sagittal	0.9°	3.9°	-6.7°/8.5°	-0.9°	3.6°	-8.0°/6.2°
	
	TO	sagittal	3.5°	3.3°	-3.0°/10.0°	1.5°	3.4°	-5.2°/8.2°

Positive results represent everted (frontal plane), abducted (transverse plane) and dorsiflexed positions

**Table 4:** Significant differences between the PPH groups in ROM for the ankle/subtalar joint complex and midtarsal joint (p ≥ 0.05)

Joint/Complex	Gait event	Cardinal Body Plane	Group 1 (PPH 2, 3 and 4)			Group 2 (PPH 1 and 5)		
			
			mean	St. Dev	95% CI (upper/lower)	mean	St. Dev	95% CI (upper/lower)
Leg/heel	HC to FF	frontal	5.1°	2.2°	0.8°/9.4°	4.0°	1.5°	1.1°/6.9°
	
	FF to AN	sagittal	9.1°	2.5°	4.2°/14°	6.8°	3.4°	0.1°/13.5°
	
	AN to HO	frontal	1.1°	1.8°	-2.4°/4.6°	-0.1°	1.6°	-3.2°/3.0°
		
		sagittal	6.8°	2.5°	1.9°/11.7°	9.1°	3.4°	2.4°/15.8°
	
	MTD to TO	sagittal	-6.6°	2.0°	-10.5°/-2.6°	-4.9°	2.1°	-9.0°/-0.8°

mid foot/heel	HC to FF	frontal	-4.1°	1.7°	-7.4°/-0.8°	-2.4°	1.6°	-5.5°/0.7°
	
	FF to AN	transverse	0.1°	1.4°	-2.6°/2.8°	0.9°	1.6°	-2.2°/4.0°
		
		sagittal	5.9°	2.6°	0.8°/11.0°	3.4°	2.4°	-1.3°/8.1°
	
	AN to HO	transverse	1.0°	1.3°	-1.6°/3.6°	0.2°	1.4°	-2.5°/2.9°
	
	HO to MAD	frontal	0.2°	0.8°	-1.4°/1.8°	-0.9°	1.0°	-2.9°/1.1°
	
	MTD to TO	transverse	-0.2°	1.2°	-2.6°/2.2°	0.5°	1.3°	-2.1°/3.1°
		
		sagittal	-4.5°	1.9°	-8.2°/-0.8°	-3.1°	1.9°	-6.8°/0.6°

First metatarsal/mid foot	FF to AN	frontal	-4.0°	3.1°	-10.1°/2.1°	-2.0°	2.5°	-6.9°/2.9°
		
		transverse	-4.4°	4.4°	-13.0°/4.2°	-2.4°	2.1°	-6.5°/1.7°
		
		sagittal	-3.8°	3.3°	-10.3°/2.7°	-2.2°	1.5°	-5.1°/0.7°
	
	MTD to TO	transverse	1.6°	1.3°	-1.0°/4.2°	1.0°	0.9°	-0.8°/2.8°
		
		sagittal	4.0°	1.9°	0.3°/7.7°	2.0°	2.0°	-1.9°/5.9°

1st MPJ	MTD to TO	frontal	-0.6°	2.5°	-5.5°/4.3°	-2.0°	2.4°	-6.7°/2.7°

Positive results represent eversion (frontal plane), abduction (transverse plane) and dorsiflexion movements

For the midfoot/heel, the midfoot of group 1 was more plantarflexed at heel contact (-9.3° compared to -6.2°), foot flat (-5.7° compared to -2.9°), at the time of maximum hallux dorsiflexion (-10.8° compared to 2.9) and toe off (-15.3° compared to -11.1°). The mid foot was also more inverted relative to the heel at foot flat (-3.4° compared to -1.6°). For the first metatarsal/mid foot, in group 1 the first metatarsal was more dorsiflexed at toe off compared to group 2 (3.5° compared to 1.5°). There were no statistically significant differences in the position of the first metatarsal phalangeal joint between groups 1 and 2.

Statistically significant differences between group 1 and 2 in terms of the range of motion in the 5 phases of stance are detailed in table 4. Group 1 displayed more heel eversion motion between heel contact and foot flat (1.1° more) and between ankle neutral and heel off (1.2° more). They displayed more dorsiflexion between foot flat and ankle neutral (2.3° more), but less dorsiflexion between ankle neutral and heel off (2.3° less). Group 1 displayed more heel plantarflexion between maximum hallux dorsiflexion and toe off (1.7° more).

For the midfoot/heel, group 1 displayed a greater range of inversion between heel contact and foot flat (1.7° more), more dorsiflexion between foot flat and ankle neutral (2.5° more) and more plantarflexion between maximum hallux dorsiflexion and toe off (1.4° more). For the first metatarsal/mid foot, group 1 displayed a greater range of inversion (2.0° more), adduction (2.0° more) and plantarflexion (1.6° more) between foot flat and ankle neutral. The only statistical difference at the first metatarsal phalangeal joint was less inversion of the hallux in group 1 between maximum hallux dorsiflexion and toe off (1.4° less). All the statistically significant differences in the ROM data (table 4) correspond to the statistically significant differences in angular values at the seven specific gait events (table 3). In addition, the ROM data can also be affected by the time at which the gait events occurred.

The timing of ankle neutral was significantly later in group 1 (36.4% vs. 31.1%, p = 0.02), and maximum ankle dorsiflexion occurred earlier (76.3% vs. 79.4%, p = 0.01) (Table 5). The total time between ankle neutral and maximum ankle dorsiflexion was therefore 8.4% of stance less in group1.

**Table 5:** Mean and standard deviation of the stance phase timing events for each of the PPH groups, and the ANOVA showing significant differences between the PPH groups (p ≥ 0.05*)

Timing event	Group 1 (PPH 2, 3 and 4)		Group 2 (PPH 1 and 5)		ANOVA		
	
	Mean	SD	Mean	SD	p value Side	p value PPH group	Covariance
FF	8.8	1.7	8.0	1.8	0.977	0.127	0.679

AN*	36.4	7.8	31.1	7.8	0.712	0.023	0.597

HO	63.3	5.7	66.6	6.3	0.703	0.064	0.718

MAD*	76.3	4.8	79.4	3.9	0.638	0.014	0.664

MTD	95.8	1.0	96.2	1.3	0.065	0.169	0.852

## Discussion

Overall the patterns and direction of movement in both groups of subjects were very similar (figures 3, 4, 5 and 6). The 95% CI (table 3 and 4) indicate that the kinematic data from feet in one group were often common to that of the other group. In the clinical paradigm of foot function the mobile and rigid foot types and their associated PPH patterns are supposed to exhibit quite distinct foot kinematic data. The lack of gross and consistent differences in kinematic data between the feet in each group is contrary to the current clinical paradigm of foot function. From this we conclude that classification of foot type (mobile, rigid) using the pattern of forefoot PPH lesions and making assumptions regarding foot kinematics based on this classification is unreliable.

Whilst the kinematic data failed to identify distinct differences between these two groups of subjects, there were several subtle (generally <3°) differences in kinematic data between the two groups. This was in both the position of the foot segments (table 3), which is sensitive to differences between groups in the position of the foot when in relaxed standing (used to set the 0 degrees position), and the data describing the range of motion between segments (table 4), which is sensitive to the timing of gait events used to define the range of motion data. According to the paradigm group 1 (associated with the mobile foot type) should display a more pronated foot, greater heel eversion, a lower medial arch, and greater first metatarsal dorsiflexion, with subsequently less hallux dorsiflexion. In fact group 1 (figure 3) displayed a less everted heel, a less externally rotated abducted heel and a more plantarflexed heel compared to group 2, which is contrary to the paradigm. For the mid foot/heel segment (figure 4) the foot is less dorsiflexed throughout stance, which might be associated with a higher medial arch compared to group 2, again contrary to the paradigm. The first metatarsal sagittal plane motion relative to the mid foot segment (figure 5), and the more dorsiflexed position of hallux between HO and MTD (figure 6) are also all contrary to the clinical paradigm. However, it should be remembered that these data only describe the position of the foot in each group relative to the position of the feet during normal standing (which was used to define the 0° position). This is not the same as stating that the foot bones and joints are actually more pronated, everted and so on, since the position of the bones under the skin is not known and the position of the bones in relaxed standing is not known. What these data describe, therefore, are differences between the two groups in the relationship between the movement of the foot joints in stance and the position the same joints adopt when stood relaxed.

The range of motion data (table 4) indicates that group 1, which the paradigm associates with a more mobile foot type, did display greater motion during stance. Of the statistically significant differences between the two groups (table 4), 72% indicated greater movement in the group 1 compared to group 2 (13 of 18 differences). However, differences were generally small in absolute terms, all were < = 2.5°. The clinical importance of such small differences is unknown and the 95% CI indicates considerable commonality in the kinematics of individual feet in each group. Thus, whilst there is evidence for greater mobility within group 1, as the clinical paradigm suggests, the nature and extent of the greater mobility is not sufficient to warrant classification of the feet we studied as consistently or distinctly more 'mobile'.

A critical part of the mobile foot type (group 1) paradigm is the assumed greater dorsiflexion of the first metatarsal in response to load under the medial forefoot and a resultant reduced hallux dorsiflexion in late stance. In both groups, the first metatarsal underwent a small amount of plantarflexion motion after forefoot loading, with the metatarsal of group 1 plantarflexing more than that of group 2 (figure 5). After the time of maximum ankle dorsiflexion the first metatarsal in group 1 displayed more plantarflexion motion than in group 2 (figure 5), which is contrary to the clinical paradigm. Furthermore, the first metatarsal phalangeal joint in group 1 shows a position of greater dorsiflexion during propulsion, which also conflicts with the clinical paradigm. Again, it should be remembered that this relates only to its position relative to its position during standing which was used to set 0°. Another theory of foot motion [28,29] suggests that the foot with greater hallux dorsiflexion during stance would be associated with a less pronated rearfoot, and this was the case for group 1 compared to group 2. However, in all these cases the actual differences in motion are small (figure 3) and none were statistically significant.

Root's paradigm proposes that the subtalar joint passes through its neutral position in the middle of stance (~50%) and at the same time as the tibia is vertical above the foot (in the sagittal plane), which broadly equates to AN in this study. Whilst we did not measure the rearfoot to leg angle when the STJ was in neutral, all prior reports state that the position of the heel when stood relaxed is everted relative to when the STJ is in its neutral position [2]. Since neither group was in an inverted rearfoot position at 50% of stance (i.e. more inverted than when stood relaxed), it is seems inconceivable that the subtalar joint was in its neutral position in the middle of mid stance, or when AN occurred. Furthermore, AN did not consistently occur at the middle of stance nor coincide with an inverted rearfoot position. Within the data presented in this study the leg and foot never simultaneously assume Roots 'neutral stance position' at any point in the stance phase of gait. It therefore seems unlikely that examination of the foot based on placing the STJ in neutral when a patient is stood upright (as proposed by Root) offers a valid representation of dynamic function. This adds further to the existing evidence that static evaluation of the foot does not reflect dynamic foot function [22,30-33].

Root's paradigm suggests that transverse plane rotation of the lower leg drives supination of the subtalar joint from the middle of midstance to just after heel off, creating a so-called 'rigid lever' for efficient propulsion. The results of this study clearly show that from AN to TO the rearfoot, forefoot, first ray and hallux are not rigid and that these foot segments are moving relative to each other. The flaw in the prior assumption by Root was that the ankle was the sole provider of the required plantarflexion. If it was, it might well require a rigid foot to effectively apply load to the ground for propulsion. These kinematic data demonstrate that many articulations in the foot contribute to the plantarflexion required to move the body forwards.

Group 2 displayed a more everted and abducted heel relative to the leg, and more dorsiflexed mid foot to heel position (table 3, figure 4). Thus foot pronation was associated with earlier ankle neutral (heel to leg = 0°) and a later time to peak heel/leg dorsiflexion. Though not statistically significant, this was also associated with less hallux dorsiflexion (figure 3). These results concur to some degree with those of a previous pilot study [34] which reported that loss of hallux dorsiflexion (induced using a rigid insole) was associated with later peak in heel/leg dorsiflexion (a prolonging of ankle dorsiflexion). Since the CI for both groups is high these differences are not definitive of each group.

There are several reasons why the foot kinematics we measured and the foot kinematics described in the clinical paradigm might not be strongly associated with the pattern of PPH. Callus develops under the metatarsal heads in response to load, and thus motion of individual metatarsals, and the motion of other bones within the foot will influence the extent to which the kinematics we measured are associated with forefoot plantar loading. For example, for heel/leg kinematics to be strongly associated with, or even predictive of, forefoot loading patterns, all other structures between the rearfoot and metatarsal heads would need to be rigid, or have predictable mechanical characteristics. There is good evidence that mid foot and metatarsal bones are capable of considerable motion and that this varies between subjects [35-37]. Other mechanisms will also influence the extent to which bone kinematics are associated with forefoot loading and PPH patterns. Hamel et al [38] described how toe flexion assisted by muscle action and plantar fascia forces influences load distribution between the toes and forefoot. Sharkey et al [39] had earlier shown how plantar fascia release altered forefoot load distribution. It follows that for two feet with the same foot kinematics, differences in the influence of the toe flexors, plantar fascia and other plantar soft tissues could result in different forefoot loading patterns. A further issue is that we used the presence of PPH to indicate forefoot pressure because this was an integral part of the clinical paradigm we sought to pragmatically investigate. However, the threshold at which PPH formation is triggered might vary between different people. Thus, the presence of PPH might reflect a low threshold to skin loading rather than a specific foot kinematic pattern that results in specific forefoot loading characteristics.

## Conclusions

There was some evidence of small differences in foot kinematics between subjects with PPH under metatarsal heads 1 and 5, and subjects with PPH under metatarsal heads 2 and 3. Though small the differences are large as a percentage of the motion occurring. There was a clear difference in the time of ankle neutral (AN) and maximum ankle dorsiflexion (MAD) between groups. The significance of these differences is unknown and there was too much similarity between the kinematic data displayed in each group to classify them as distinct foot types as the current clinical paradigm proposes. PPH lesion distribution is a poor basis for classification of foot kinematics.

## Competing interests

There are no financial competing interests (political, personal, religious, ideological, academic, intellectual, commercial or any other) to declare in relation to this manuscript

## Authors' contributions

AHF conceived, designed and carried out the kinematic studies; performed the statistical analysis and drafted the manuscript.

CJN helped in the design of the kinematic studies and to draft the manuscript.

PB helped in the design of the kinematic studies.

All authors have read and approved the final manuscript.
